# A Model Abattoir at Liverpool

**Published:** 1924-10

**Authors:** 


### A MODEL ABATTOIR AT LIVERPOOL.

The Liverpool City Council has approved the general plans of the new abattoir scheme which has been formulated by the Market Committee. The Report of the Clerk and Superintendent of Markets, Mr. A. D. Harper, O.B.E., gives some interesting details as to the construction of the proposed abattoir which Mr. T. Laurie Price has designed. All the modern methods are provided for, and the scheme for the slaughter of animals will eliminate the existing unavoidable cruelty in taking them from the lairage to the point of slaughter. The lairage accommodation is to be situated in every case immediately behind the slaughter stations, and animals will be driven into a waiting pen near the actual place of slaughter, and will be entirely obscured from the view of other animals being slaughtered. The method of slaughter is that of the stunning pin.

### Keeping the Abattoir Clean.

A point which necessitated considerable attention was a means of satisfactorily disposing of the offal, paunches, feet, skins and hides, which form an objectionable feature in every abattoir in the country. This has been overcome by a system of chutes, which will convey the objectionable matter to a room below, where it will be dealt with by the offal and other contractors. After preliminary treatment, the hides and skins, etc., will be taken along an offal corridor to the rear of the building to premises assigned to the respective contractors. The blood is to be disposed of by gush pits, and thence conveyed to a central blood vat, from which it will be pumped to the blood factory at the rear of the abattoir. By this means the whole of the objectionable features of the abattoirs of this country will be eliminated ; the floors will be kept clear of garbage, and only edible meat will be seen in the slaughter places. After the beasts are dressed they will be conveyed by rail into a natural air-cooling room, the accommodation of which is, in each case, equivalent to a full day's killing.

### Disposing of the Meat.

The meat can be loaded up from the cooling room for road transport, or directly on to a loading platform to the railway trucks situated conveniently to the cooling rooms. The meat can similarly be despatched by a continuous rail into the meat market, or, alternatively, lowered on the rails to the chill rooms underneath the meat market. Two new lines will be provided for the incoming frozen and chilled meat, which can be taken into cold store by means of lifts on the unloading platform, and thence, by means of further lifts, to the market proper as and when required.

### The Unmarried Mother.

The Annual Report of the National Council for the Unmarried Mother and her Child states that the Case Committee has dealt with nearly 600 applications, and the usefulness of this department steadily increases. This work is very valuable but exceedingly difficult, involving many interviews and much correspondence. The Executive Committee has drafted a Bill dealing with affiliation orders, which it hopes to submit in the autumn to the Council and to the Joint Legislative Committee of interested bodies, with a view to its introduction next year.

